# Computational Biomechanics: In-Silico Tools for the Investigation of Surgical Procedures and Devices

**DOI:** 10.3390/bioengineering7020048

**Published:** 2020-05-30

**Authors:** Emanuele Luigi Carniel, Ilaria Toniolo, Chiara Giulia Fontanella

**Affiliations:** Department of Industrial Engineering, Centre for Mechanics of Biological Materials, University of Padova, I-35131 Padova, Italy; emanueleluigi.carniel@unipd.it (E.L.C.); ilaria.toniolo.1@phd.unipd.it (I.T.)

**Keywords:** computational methods, constitutive model, dental implantology, surgery

## Abstract

Biomechanical investigations of surgical procedures and devices are usually developed by means of human or animal models. The exploitation of computational methods and tools can reduce, refine, and replace (3R) the animal experimentations for scientific purposes and for pre-clinical research. The computational model of a biological structure characterizes both its geometrical conformation and the mechanical behavior of its building tissues. Model development requires coupled experimental and computational activities. Medical images and anthropometric information provide the geometrical definition of the computational model. Histological investigations and mechanical tests on tissue samples allow for characterizing biological tissues’ mechanical response by means of constitutive models. The assessment of computational model reliability requires comparing model results and data from further experimentations. Computational methods allow for the in-silico analysis of surgical procedures and devices’ functionality considering many different influencing variables, the experimental investigation of which should be extremely expensive and time consuming. Furthermore, computational methods provide information that experimental methods barely supply, as the strain and the stress fields that regulate important mechano-biological phenomena. In this work, general notes about the development of biomechanical tools are proposed, together with specific applications to different fields, as dental implantology and bariatric surgery.

## 1. Introduction

Cooperation among clinicians and bioengineers has historically proved its efficacy and strength by providing tools, devices and methodologies for the optimization of health-care systems, devices, procedures and approaches [1]. With specific regard to the field of surgery, the combination of the typical rationalism of engineers and the creativity of surgeons led to novel and unimaginable evolutions and solutions [2,3]. Nowadays, a challenge pertains to the 3R approach, as reduce, refine and replace, to the use of animal models for scientific purposes and pre-clinical research. The 3R approach greatly influences common procedures for the definition, the design, the optimization, the reliability assessment and the certification of surgical procedures, instrumentations and devices. Aiming at this ethical and mandatory 3R approach, the competences and the skills of bioengineers can play a meaningful role [4]. Computational biomechanics provides structural modeling tools that allow for simulating the mechanical behavior of anatomical regions and the interaction phenomena occurring among such biological structures and surgical instrumentations [5] and/or prosthetic devices [6]. The accuracy and the reliability of computational methods continuously improve, because of the advancements in modelling research and the increase in computational power. In this sense, in-silico analyses and simulations can actually replace, reduce and refine experimentations on animal models [7].

**Figure 1:**
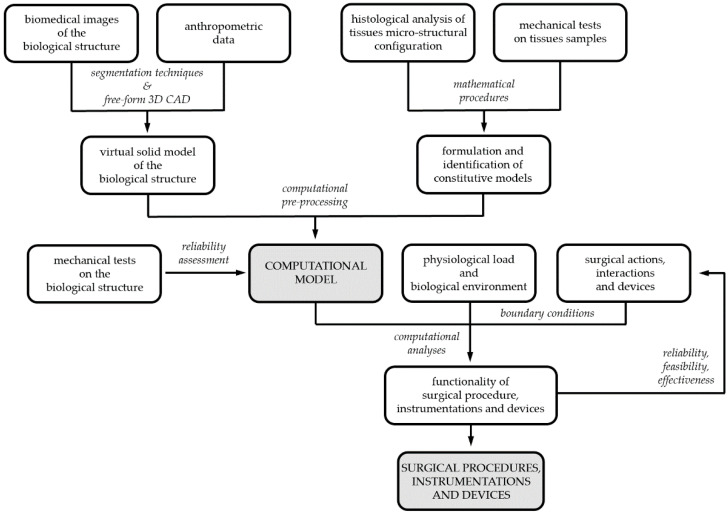
Biomechanical models and computational techniques for surgery: coupled experimental and computational activities for model’s definition, identification and validation; model exploitation for designing, optimizing and certifying surgical procedures, instrumentations and devices.

Developing the biomechanical model of a biological structure requires the characterization of both the geometrical conformation of the anatomical district, by means of virtual modelling techniques, and the mechanical behavior of building tissues, by means of constitutive investigations. Coupled experimental and computational activities are usually adopted to reach such a goal (Figure 1) [8]. Experimentations are mandatory for models’ definition, identification and reliability assessment [9]. Biomedical images from tomographic techniques, such as CT and/or MRI, together with anthropometric information lead to 3D CAD virtual models, whose discretization by finite element pre-processing software provides the geometrical definition of the computational model. The constitutive analysis of biological tissues requires information about both tissues micro-structural configuration, from histological investigation, and tissues’ mechanical behavior, from experimental tests on tissue samples. Finally, the assessment of computational model reliability requires comparing model results and data from further experimentations, which must be performed on the entire biological structure. According to the 3R approach, experimentations can be frequently performed on samples from animal models, as wastes from local abattoirs, and human surgical scraps.

Despite the relevant experimental effort that is required for defining the computational model, the model allows for investigating the biological structure mechanics, assuming an extremely broad range of conformations of the anatomical district, which correspond to many different patients, and of loading situations, which allow for describing many different surgical interventions. Furthermore, computational methods allow for achieving information that experimental methods barely supply, such as strain and stress. The evaluation of such mechanical stimuli is a relevant task, because they regulate many different phenomena: mechanical aspects, such as tissue damage or breakage, physio-mechanical phenomena, such as tissue remodeling and adaptation, mechano-biological aspects, such as transduction of mechanical stimuli to other signals, etc.

## 2. Overview on Computational Modeling of Biological Structures 

The biomechanical model of a biological structure aims at describing its mechanical behavior, as the deformation of the structure when mechanical loads act on the structure itself. Fundamental balance principles, such as the conservation of mass, equilibrium of forces and equilibrium of momenta, govern such evolution by means of partial differential equations. External loads and constraints specify the boundary conditions of the problem [10]. The complexity of the problem usually denies the analytical approach, and numerical methods, as the finite element method, must be adopted [11,12]. Practically, numerical methods transpose the differential problem into an algebraic problem, which is suitable for computational implementation and processing.

The development of the computational model of a biological structure requires the geometrical definition, which is achieved by means of virtual solid and finite element models, and the mechanical characterization of the building tissues by means of constitutive formulations. Boundary conditions specify the surgical actions by means of boundary conditions. Moreover, the specific analysis techniques and computational strategies must be addressed.

## 3. Geometrical Characterization of Biological Structures

Three-dimensional virtual solid models of biological structures are usually developed by processing images from tomographic or histo-anatomical techniques together with anthropometric data. CT and MRI are typical tomographic technologies, which capture contiguous series of image slices. A slice defines a cut through the scanned biological structure and different slices are performed according to a settled thickness. The pixels of the image slice provide measurements of local material properties. Regions of homogeneous pixel values usually represent the inner portions of anatomical elements, whereas strong variations of pixel values identify tissue boundaries. Concatenating image slices leads a three-dimensional image stack, and anatomical elements can be traced between adjacent images. Virtual solid models of anatomical elements are extrapolated by means of segmentation procedures and 3D CAD free-form operations [13].

The next step of geometrical modelling pertains to finite element discretization, the fundamental issues of which are element topology and mesh density [12]. Mesh density is usually a compromise between the accuracy of results and computational effort. Non-uniform mesh density is frequently assumed; refined finite element discretization may be provided considering specific regions of the model, where the geometry is particularly complex or a more accurate solution is required [14].

## 4. Constitutive Analysis of Biological Tissues’ Mechanics

The constitutive model defines the mechanical response of a material by means of mathematical relationships, which specify the stress that the material experiences in terms of other field functions, such as the strain history [10,15]. The constitutive analysis of a biological tissue requires experimentally investigating both the microstructural arrangement and the mechanical behavior [8]. Complex and hierarchical microstructural configurations characterize biological tissues, the organizations of which are optimized to fulfill their specific mechanical functions. Microstructural rearrangement processes establish the phenomenological mechanical behavior of the material, as the response that the material exhibits during mechanical tests and, more generally, during mechanical loading. Microstructural information and data from experimental activities suggest the mathematical formulation of the constitutive model, which must be defined in the framework of the axiomatic theory of constitutive laws. The axiomatic theory provides for the following general rules [10]:

(1) $$S\left(E,A^{i};p\right)=\frac{\partial \psi \left(E,A^{i};p\right)}{\partial E}$$

(2) $$G\left(A^{i},{\dot{A}}^{i};p\right)=0$$

(3) $${\dot{d}}_{\mathrm{int}}=-\sum_{i}\frac{\partial \psi \left(E,A^{i};p\right)}{\partial A^{i}}:\;{\dot{A}}^{i}\geq 0$$

where ***E*** and ***S*** are appropriate and corresponding strain and stress tensors, respectively. Material straining entails energy consumption, as the work of the stress. Such energy is partially stored into the material in a recoverable form; the material will give it back in the form of mechanical work. The remaining part of the work of the stress is dissipated. From a microstructural point of view, material straining leads to modifications of the microstructural configuration and energy adsorption. Such microstructural modifications may be reversible, leading to recoverable energy storage; otherwise, irreversible modifications entail energy dissipation. Furthermore, friction phenomena may occur during microstructural modifications, providing further energy dissipation in heat. The development of dissipative phenomena, as the irreversible microstructural modifications and the friction effects, is described by means of internal variables ***A****^i^*. From a phenomenological point of view, the internal variables may describe plastic strains, damage effects, non-equilibrated viscous stress components, etc. The Helmholtz free energy *ψ* evaluates the recoverable stored energy, which is a function of the material strain history in terms of the strain ***E*** and the internal variables ***A****^i^*. Differentiation operations lead to the stress–strain relationship (Equation (1)). Algebraic differential equations ***G*** specify the evolution of internal variables with the strain history (Equation (2)). The Helmholtz free energy *ψ* and the evolution equations ***G*** must satisfy the dissipation rule (Equation (3)), which states that the energy dissipated during the strain history, as *d*_int_, has to increase or remain constant. 

A particular class of constitutive equations—a class that includes elastic and, more precisely, hyperelastic formulations—assumes the absence of dissipative phenomena (*d*_int_ remains constant). All the work of the stress is recoverably stored and is evaluated by the Helmholtz free energy. According to the dissipation rule (Equation (3)) and to the stress–strain relationship (Equation (1)), the Helmholtz free energy *ψ* and the stress ***S*** are functions of the strain ***E*** only; the Helmholtz free energy *ψ* is a potential of the strain ***E*** and is denominated strain energy function *W*. Otherwise, the dissipated energy *d*_int_ increases during the strain history, and the material is said to have inelastic or dissipative behavior. 

The vector of constitutive parameters ***p*** is related to the properties of the specific material (i.e., elastic constants, viscous parameters, yielding and hardening constants, damage limits, etc.). Once the model mathematical formulation has been defined, the next step of the constitutive analysis pertains to the identification of such parameters. The action is usually based on an inverse analysis by assuming a stress–strain history given by experimental data, designed at the purpose, and estimating the parameters that yield the best fit with analytical or computational results based on the assumed constitutive model. Aiming at the univocal identification of parameters, the required experimental situations must be accurately defined [3].

### 4.1. Hard Tissues

A highly hierarchical micro-structural organization characterizes bone tissue, the principal constituents of which are both organic (collagen fibers, non-collagenous proteins and cells) and inorganic (hydroxyapatite crystals). Two principal types of bone tissue compose bony structures, as cortical bone, which is a dense and compact mass, and trabecular bone, the typical feature of which is the reticular conformation. Cylindrical structures, known as osteons, compose cortical bone, while trabecular bone is a lattice of trabeculae. A cylindrical wrapping of bony lamellae builds up osteons and trabeculae. Each lamella is a fiber-reinforced plate, with collagen fibers embedded within a ceramic matrix of hydroxyapatite. Within, the lamella fibers run in approximately the same direction, but the axes of these fibers can differ by as much as 90° in adjacent lamellae. Bone constitutive formulations are frequently defined in the framework of linear elasticity [16]:

(4) σ(ε)=D:ε

where **σ** is the true stress tensor (Cauchy stress tensor), **ε** the small strain tensor and ***D*** the fourth rank elasticity tensor. The microstructural configuration of bone tissues entails the anisotropic behavior, because of the orientation of osteons and trabeculae along major stiffness directions. Transversally isotropic and orthotropic formulations of the elasticity tensor ***D*** have been frequently adopted, which require the identification of five or nine independent elastic parameters, respectively. Parameters’ identification requires results from mechanical tests, which can be performed on tissue specimens from different bony structures, considering both animal models and human samples [16], or processing CT data by means of relationships between Hounsfield Unit values and elastic parameters [17]. 

A more accurate characterization of bone mechanics requires investigating dissipative phenomena, which leads to nonlinear formulations of the problem [18]. Viscoelastic behavior occurs because microstructural phenomena (i.e., mutual sliding of osteons or lamellae, etc.) need time to develop. Viscoelastic models allow for interpreting the resulting time-dependent effects, such as strain rate sensitivity, hysteresis, stress relaxation and creep:

(5) $$\sigma \left(\varepsilon ,t\right)=D:\varepsilon -\sum_{i}Q^{i}\left(\varepsilon ,t\right)$$

(6) $${\dot{Q}}^{i}+\frac{1}{\tau^{i}}Q^{i}=\frac{\gamma^{i}}{\tau^{i}}D:\varepsilon$$

where viscoelastic internal variables ***Q****^i^* specify non-relaxed stress components, while *γ^i^* and *τ^i^* are associated viscoelastic parameters (the relative stiffness and the relaxation time, respectively). Differential equations (Equation (6)) provide the evolution criterion of viscoelastic internal variables [15]. 

The excessive loading of bony structures may induce irreversible microstructural modifications, leading to permanent strain. Aiming to interpret such inelastic behavior, elastoplastic formulations must be assumed:

(7) $$\sigma \left(\varepsilon ,\varepsilon_{pl}\right)=D:\left(\varepsilon -\varepsilon_{pl}\right)$$

(8) $$\phi \left(\varepsilon ,\varepsilon_{pl},\dot{\varepsilon },{\dot{\varepsilon }}_{pl}\right)=0$$

Where the plastic internal variable **ε***_pl_* specifies permanent strain components, while the yielding criterion *φ* (Equation (8)) governs the development of permanent strains [18].

### 4.2. Soft Tissues

Soft biological tissues are collections of cells and an Extra Cellular Matrix (ECM). Connective tissues are characterized by a few cells embedded within an abundant ECM, which is responsible for their mechanical behavior. Examples of soft connective tissues are tendons, ligaments and cartilage. Cellular tissues are mainly composed of cells and the minimal ECM serves to separate cells or groups of cells. Muscular, nervous and epithelial tissues are typical examples.

The microstructural configuration of soft biological tissues suggests the assumption of fiber-reinforced composite material. Fibrous elements, such as collagen, elastin, muscle and/or nervous fibers, are embedded in an isotropic ground matrix, and their orientation determines the strongly anisotropic behavior of soft tissues. Tissue strain involves the microstructural rearrangement phenomena of both fibers and ground matrix, such as fibers’ uncrimping and the flux of liquid phases. Such phenomena entail the typical variation of material stiffness with stretch, the mathematical description of which is usually performed by means of hyperelastic formulations [19]. Defining a hyperelastic model requires one to specify the strain energy function *W* [10]:

(9) $$P\left(F,M_{j}\right)=\frac{\partial W\left(F,M_{j}\right)}{\partial F}$$

(10) $$W\left(F,M_{j}\right)=W^{m}\left(F\right)+\sum_{j}W_{j}^{f}\left(F,M_{j}\right)$$

where ***P*** and ***F*** are the nominal stress tensor (first Piola-Kirchhoff stress tensor) and the deformation gradient, respectively. The composite configuration of soft biological tissues suggests splitting the strain energy function into an isotropic contribution of the ground matrix *W^m^* and anisotropic contributions of fiber families *W_j_^f^* (Equation (10)). The fabric tensor ***M****_j_* is associated with the local orientation of the *j*^th^ fiber family [8]. Many different hyperelastic models of soft biological tissues have been proposed in the scientific literature [20]. 

Microstructural rearrangement phenomena require significant time to develop, leading to the typical time-dependence of soft biological tissues mechanical behavior. Visco-hyperelastic models allow for analyzing such dependence [21,22]:

(11) $$P\left(F,M_{j},t\right)=\frac{\partial W\left(F,M_{j}\right)}{\partial F}-\sum_{i}Q^{i}\left(F,M_{j},t\right)$$

(12) $${\dot{Q}}^{i}+\frac{1}{\tau^{i}}Q^{i}=\frac{\gamma^{i}}{\tau^{i}}\frac{\partial W\left(F,M_{j}\right)}{\partial F}$$

where viscoelastic internal variables ***Q****^i^* specify non-relaxed stress components, while *γ^i^* and *τ^i^* are associated viscoelastic parameters [15].

### 4.3. Identification of Constitutive Parameters

The constitutive analysis of biological tissues requires both the mathematical formulation of the model and the identification of the associated parameters. Constitutive parameters’ identification is challenging because of the complexity of biological tissues’ mechanical behavior, the difficulty of biological samples’ management, and the significant inter-specimen variability. Inverse analyses techniques are usually exploited for parameters identification on the basis of data from experimental activities [8,23]. 

The planning and design of mechanical tests is a crucial step. Mechanical tests should be performed considering different loading conditions, such as tensile, compressive and shear, and different loading directions. In more detail, the selection of mechanical tests depends on the constitutive formulation assumed and, consequently, on the parameters that must be identified. In fact, mechanical tests must disclose all the mechanical properties that are associated with the assumed constitutive parameters. Otherwise, parameters identification leads to non-univocal results. A refined constitutive formulation, which perfectly interprets all the details of the tissue mechanics, usually involves many different parameters, which are associated with the different mechanical features of the tissue. The univocal identification of the parameters requires an extensive experimental campaign. A simpler constitutive model could interpret the tissue mechanics with lower accuracy. On the other side, fewer parameters must be identified, requiring lower experimental effort. It follows that constitutive model complexity is usually a compromise between the accuracy of the tissue characterization and extent of the required experimental campaign.

Predictive models interpret the experimental situations by considering the constitutive formulation and the specific loading conditions of the developed mechanical tests. The predictive model provides relationships between model values of the stress, such as *S*_mod_, the applied strain conditions *E* and the assumed constitutive parameters ***p***:

(13) $$S_{\mathrm{mod}}=S_{\mathrm{mod}}\left(E;p\right)$$

The predictive model can be defined by means of analytical or computational formulations. Model stress values *S*_mod_ are compared with experimentally measured values, such as *S*_exp_, in consideration of all the *q* strain conditions investigated *E*_z_. A cost function evaluates the overall discrepancy between experimental data and predictive model results:

(14) $$\Omega \left(p\right)=\sqrt{\frac{1}{q}\sum_{z=1}^{q}{\left(1-\frac{S_{\exp}\left(E_{z}\right)}{S_{\mathrm{mod}}\left(E_{z};p\right)}\right)}^{2}}+\Theta \left(p\right)$$

where Θ is a penalty contribution that evaluates the satisfaction of material stability requirements [24]. A small value of the cost function represents a valid agreement between experimental and model results and, consequently, appropriate constitutive parameters. The minimization of the cost function leads to the optimal set of parameters. Frequently, the complexity of the constitutive formulation entails multimodal behavior of the cost function. Coupled stochastic and deterministic algorithms must be assumed aiming at an efficient procedure for the minimization problem [25]. Finally, data from further independent mechanical tests are required to assess the reliability of the identified parameters.

## 5. Computational Analysis Techniques

Biomechanical investigations aim at analyzing the response of the biological structure when specific actions are applied. Boundary conditions define such actions, together with motion constraints and interaction phenomena among the biological structure and surrounding environment and/or objects. 

The biomechanical analysis may concern dynamic or quasi-static problems [11]. A dynamic approach is required if significant inertial effects occur, otherwise static procedures are suitable. A further issue pertains to the linearity or nonlinearity of the problem. Linear problems involve small displacements and strains, linear constitutive relationships, and linear boundary conditions. Otherwise, the mechanical problem is nonlinear. The numerical formulation of the problem entails the transposition of a differential problem to the solution of algebraic equations. The linearity or nonlinearity of the problem entails linearity or nonlinearity of numerical algebraic procedures. Greater computational effort characterizes nonlinear problems, because iterative solution algorithms are mandatory. Finally, the biomechanical problem may involve other physical phenomena, such as fluid dynamics, thermal, electromagnetic and/or diffusion processes. Multiphysics techniques provide coupled approaches in the numerical solution of such multiple interacting physical domains.

## 6. In Silico Analysis of Surgical Procedures: Case Studies

Aiming to better depict the capabilities of computational methods in the field of surgery, typical situations are reported. The proposed investigations analyze both surgical procedures and devices, taking account of the topics of both hard and soft tissue mechanics.

### 6.1. Biomechanical Tools for the Reliability Assessment of Multi-Implant Systems in Dental Implantology

Dental implantology represents the gold-standard treatment for partial or total edentulism. Many different factors determine the success of the intervention and pertain to clinical, biomechanical, biomaterials and manufacturing aspects. One of the principal topics concerns the mechanical stimulation of bone tissue by the prosthetic system. Excessive bone stressing induces bone damage and necrosis, while insufficient stimulation leads to bone resorption. Implant failure occurs in both the situations. In this sense, computational methods provide an excellent and incomparable framework for the reliability and effectiveness investigation of surgical devices and procedures. In fact, biomechanical modelling entails the evaluation of stress and strain fields within bone tissues depending on many different influencing variables. On the other side, the reliability investigation by means of experimental techniques would require an extremely expensive campaign on animal models. 

**Figure 2:**
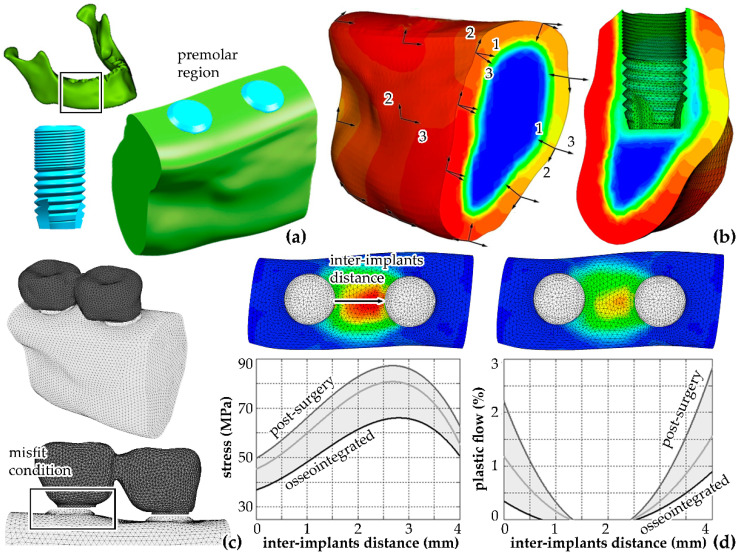
Virtual solid models: the mandible, the implant and the implants’ insertion within the premolar region (**a**). The identification of the distribution of orthotropic elastic constants and direction from CT data (contours of the Young modulus along the distal–mesial direction, ranging between 0 and 21 GPa) (**b**). The finite elements model of the system and misfit condition (**c**). The results from computational analyses (plots and contours of compressive stress, ranging between 0 and 90 MPa, and plastic flow, ranging between 0% and 3%) (**d**).

The main challenging situations concern multi-implant systems, because the coupling between implants and connecting bar can induce misfits, which may determine significant and unexpected stress and strain phenomena. It follows that the manufacturing of such coupling bars must be precisely defined to ensure the reliability of the overall surgical intervention. Biomechanical methods allow detailed investigations of misfit phenomena, and specific computational models have been developed [26]. In detail, models allow for evaluating the stress–strain field intensity and its short-term evolution in the peri-implant bone tissue depending on many different influencing variables, such as bar conformation, implants’ properties, bone quality and bone–implant interface. CT data processing led to a virtual solid model of the human mandible. The premolar region was extracted, and solid models of implants and the connecting bar were virtually inserted according to a configuration that is frequently adopted in clinical practice (Figure 2a). Because misfit can induce the development of permanent strains in the peri-implant region and the stress–strain field usually evolves in time, the reliable investigation of cortical and trabecular bone tissues’ mechanics required an orthotropic visco-elasto-plastic constitutive model [18]. The analysis of CT data further allowed for evaluating the non-homogeneous distribution of orthotropic elastic properties and directions [27]. With regard to the peri-implant region, the definition of local bone and bone–implant interface properties made it possible to analyze different osseointegration conditions, from the immediate post-surgery condition to the fully osseointegrated state (Figure 2b). Specific boundary conditions were introduced to define the misfit condition (Figure 2c). The computational results reveal relevant strain phenomena in the inter-implants region, which lead to permanent strain effects. The specific properties of peri-implant tissue and bone-implant interface significantly influence the magnitude and the distribution of the stress–strain fields (Figure 2d).

### 6.2. Biomechanical Models for the Computational Evaluation of Gastrointestinal Bariatric Procedures

Obesity is one of the main health concerns worldwide, and it is estimated that about 650 million people were affected by obesity in 2016 [28]. Obesity is defined by body mass index (weight (kg)/height (m^2^)) ranging between 30 and 35 kg/m^2^, while values between 35 and 40 kg/m^2^ identify severe obesity and morbid obesity shows values higher than 40 kg/m^2^.

Bariatric surgery is the gold standard treatment for severe obesity; nevertheless, unsatisfactory weight loss and complications can occur. Most of the bariatric procedures, such as Laparoscopic Sleeve Gastrectomy (LSG), Roux-en-Y Gastric Bypass (RYGB) and Adjustable Gastric Banding (AGB), are performed laparoscopically with their inherent limitations, such as the high anesthetic dose [29]. In recent years, endoluminal interventions, such as Endoscopic Sleeve Gastroplasty (ESG), and devices have been developed with the aim to make safer and more accessible bariatric procedures, also to people with morbid obesity and obesity-related pathologies. However, the lack of standardization, the greater technical difficulty, the long-lasting and the unknown long-term efficacy have slowed down the spread of ESG techniques [30,31]. Bariatric surgery is mainly defined on experiential bases and surgeons’ practice, and a more rational approach is still challenging. Biomechanical methods can provide powerful tools in the evaluation of stomach functionality depending on bariatric procedure. Computational models make it possible to investigate a vast scenario, and novel and innovative procedures can be simulated without activating experimentations on animal models or in vivo clinical trials.

**Figure 3:**
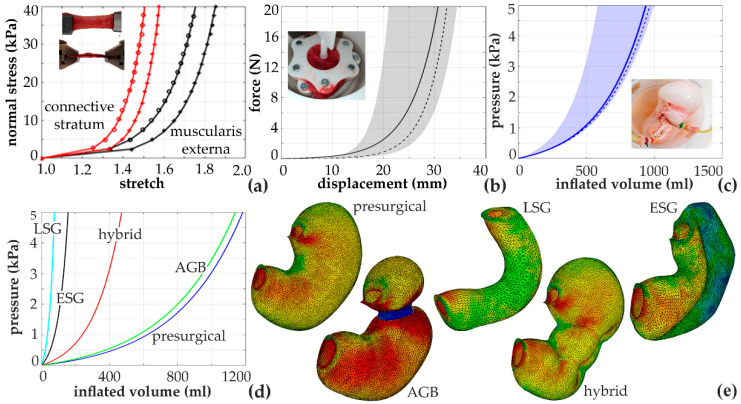
Constitutive model definition and identification of parameters by means of the inverse analysis of tensile tests on stomach tissue specimens (experimental data, as empty dots, and constitutive model results, as continuous lines) (**a**). A reliability assessment of the constitutive model and parameters by means of membrane flexural tests (statistical distribution of experimental data, as a discontinuous line and gray band, and the computational model results, as a continuous line) (**b**). The reliability assessment of the stomach finite element model by means of insufflation tests (statistical distribution of experimental data, as a discontinuous line and blue band, and the computational model results, as a continuous line) (**c**). A computational analysis of stomach functionality in pre- and post-surgical configurations: pressure-volume behavior (**d**) and maximum principal strain contours (ranging between 0% and 150%) (**e**).

A biomechanical model of the stomach was developed on the basis of coupled experimental and computational activities [5,32]. Experimentations were performed on samples from animal models, such as wastes from local abattoirs, and human surgical scraps from Padua University Hospital. Data from histological analyses and mechanical tests at tissue level, such as constant strain rate and stress–relaxation tensile tests, led to the definition and the identification of a fiber-reinforced visco-hyperelastic constitutive model (Figure 3a). The reliability of the constitutive model and parameters was assessed by analyzing independent experimentations, such as membrane flexural tests (Figure 3b). Anatomical data and anthropometric information made the geometrical definition of the stomach finite element model possible, which consisted in a double-layered structure (mucosa–submucosa connective stratum and muscularis externa), which was further divided in three main regions (fundus, corpus and antrum). Coupled experimental and computational investigations of the entire stomach mechanics, as insufflation tests, ensured the reliability of the model (Figure 3c).

The developed model was exploited to analyze stomach functionality before and after bariatric surgery, with regard to both current techniques, such as LSG, AGB and ESG, and novel approaches, such as hybrid interventions between ESG and AGB. Bariatric surgery aims at reducing stomach capacity and at anticipating meal-induced satiety, which is also mediated by the mechanical and chemical stimulation of gastric receptors [33]. Computational investigations proved their efficacy in evaluating the influence of the intervention on both pressure-volume behavior (Figure 3d), such as a measure of stomach capacity, and the stress and the strain fields that occur after food ingestion (Figure 3e), such as the mechanical stimulation of gastric receptors.

## 7. Future Perspectives and Concluding Remark

The reported investigations prove the efficacy and the effectiveness of biomechanical models for in-silico investigating surgical procedures, instrumentations and devices. Computational analyses allow for both assessing the reliability of current surgical technologies, such as procedures, instrumentations and devices, and defining and optimizing novel techniques. Activities that are required for models’ definition have been briefly described with regard to the most challenging topics, such as the geometrical modeling of anatomical districts and the constitutive modelling of biological tissues mechanics. 

Despite the relevant efforts in developing a reliable computational model, computational techniques make it possible to analyze the influence of many different surgical and patient variables. As an example, the modelling approach allows for testing different surgical techniques on exactly the same configuration of the biological district. On the other side, the experimental approach requires different samples for different experimentations, introducing the unavoidable influence of inter-sample variability. Similarly, computational methods can be exploited to analyze the influence of patients’ characteristics on surgical outcomes. The modification of model parameters allows for interpreting different patients, while the setting of loading and boundary conditions ensures exactly the same surgical intervention. On the other side, the experimental approach cannot ensure a perfect equivalence between two interventions, leading to inter- and intra-surgeon variability. 

Furthermore, computational analyses provide a quantitative definition of stress and strain distributions, which experimental activities barely provide. The strain describes the shape modification that the biological tissue locally experiences, while the stress specifies the mechanical actions that the tissue locally senses. The knowledge of such mechanical stimuli is crucial in the field of surgery because they regulate many different mechano-biological effects, such as tissue damage or failure, tissue adaptation and mechano-transduction phenomena.

In conclusion, experimentations are, nevertheless, mandatory for a model’s definition, and the subsequent computational activities allow for a broader and more accurate investigation than experimentations on animal models can provide.
